# Congenital ileal stenosis

**DOI:** 10.1093/jscr/rjag053

**Published:** 2026-02-17

**Authors:** Michael Wang, Erika B Lindholm

**Affiliations:** Department of General Surgery, Cooper University Hospital, 1 Cooper Plaza, Camden, NJ 08103, United States; Division of Pediatric Surgery, Children’s Regional Hospital at Cooper, Department of Pediatrics and Surgery, Cooper Medical School of Rowan University, 1 Cooper Plaza, Camden, NJ 08103, United States

**Keywords:** intestinal stenosis, intestinal atresia, congenital

## Abstract

Congenital jejunal-ileal atresia (JIA) is a critical neonatal diagnosis, as morbidity and mortality can be high if not treated. JIA usually presents with obstipation, and diagnosis can be established with history and physical exam, radiography, and contrast fluoroscopy. This case demonstrates ileal stenosis, a rare type of JIA, presents with intermediate obstructive symptoms. A 39-week newborn female presented with bilious emesis and dilated loops of bowel on X-ray. Contrast fluoroscopy ruled out volvulus and colonic pathology. Patient had persistent bowel function and had evolving but persistent small bowel dilation with eventual pneumatosis on Day 10. Exploratory laparotomy revealed ileal stenosis with proximal dilated bowel. She underwent resection and primary anastomosis. She recovered well and is progressing appropriately. Congenital ileal stenosis is a rare type of atresia with incomplete obstruction of the lumen resulting in equivocal obstructive symptoms. This presents a diagnostic dilemma as classical congenital atresias present with obstipation.

## Introduction

Congenital jejunal-ileal atresia (JIA) is a critical neonatal diagnosis, as morbidity and mortality can be high if not treated. Congenital ileal stenosis is a rare type of atresia with incomplete obstruction of the lumen resulting in equivocal obstructive symptoms [1]. This presents a diagnostic dilemma as classical congenital atresias present with obstipation.

## Case report

Patient is a female born at 39w4d by spontaneous vaginal delivery after an uncomplicated pregnancy. She passed meconium but subsequently developed bilious emesis and feeding intolerance at 6 h after birth. She had a soft, distended abdomen without pain on palpation.

**Figure 1:**
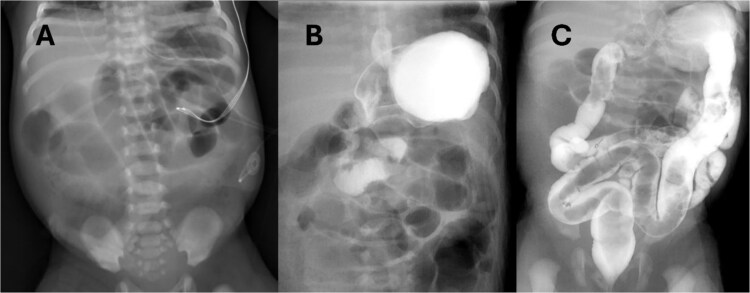
(A) AXR on DOL1 showing diffuse small bowel dilation, status post placement of replogle. (B) UGI revealing passage of contrast without evidence of malrotation or volvulus. (C) CE with normal caliber colon and no filling defect.

Abdominal radiograph (AXR) (Fig. 1) revealed dilated loops throughout the abdomen. A replogle was placed for decompression. Urgent upper gastrointestinal series (UGI) and contrast enema (CE) (Fig. 1) performed showed slightly delayed gastric emptying but otherwise low concern for malrotation, midgut volvulus, or Hirschsprung's disease. Antibiotics and parenteral nutrition were initiated.

She was closely monitored in the neonatal intensive care unit where vital signs were normal. Nasogastric decompression initially had large volume bilious output. Over the next few days, the output fluctuated between clear and bilious without change in clinical exam. Labs were grossly unremarkable without leukocytosis.

**Figure 2:**
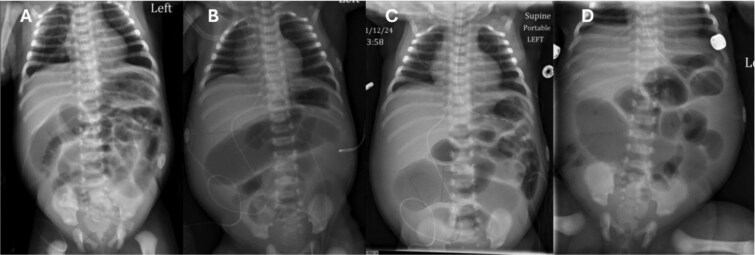
Progression of AXR. Note the changing bowel gas patterns. (A) DOL 2. (B) DOL 5. (C) DOL 6. (D) DOL 10 with concern for pneumatosis.

On day of life (DOL) 6, she was noted to have a melanotic stool with increased abdominal distension. Repeat AXR showed presumed dilated transverse colon (Fig. 2). There was concern for infectious pathology, however blood cultures, urine cultures, and cytomegalovirus serology later resulted as unremarkable. DOL 10 AXR (Fig. 2) illustrated new linear lucencies, possible pneumatosis, and patient was taken to the operating room.

**Figure 3:**
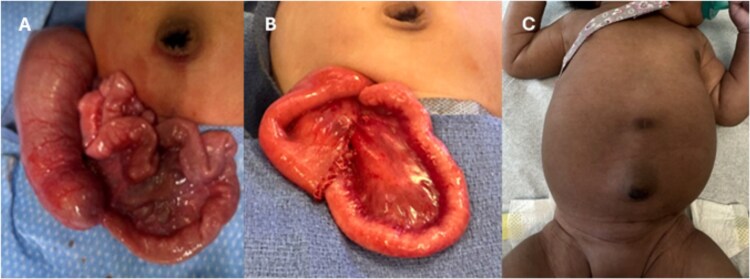
(A) Operative findings of ileal stenosis. (B) Postoperative resection with anastomosis. (C) Clinical follow-up with well healed right lower quadrant scar.

She underwent a diagnostic laparoscopy which revealed absence of abdominal wall bands. The procedure was converted to an exploratory laparotomy with incision in the right lower abdomen. The bowel was examined extracorporeally and focal narrowing with dilated bowel proximal was noted in the ileum (Fig. 3). There was mild venous congestion noted in the proximal bowel but otherwise appeared viable. This region was resected with ~10 cm of bowel proximal to the narrowing in order encompass areas of possible pneumatosis. A single layer primary anastomosis was created, followed by fascial and skin closure.

Post operatively, the patient recovered well. She continued total parenteral nutrition (TPN) with replogle to suction. She had return of bowel function on post-op day (POD) 3, and feeds were initiated on POD4. By POD6, the patient was tolerating oral feeds ad-lib. She was discharged home on POD10 in healthy condition. Clinic follow up visits revealed a well appearing baby with normal feeding and stooling. Surgical pathology revealed a segment of small bowel with luminal narrowing, focal mucosal ulceration, and mild ischemic-type changes.

## Discussion

Congenital small bowel atresia is a rare cause of newborn obstructions, occurring in ~2.3 per 10 000 live births [2]. While the most common type of atresia is duodenal atresia; jejunal-ileal atresias are the 2^nd^ most common and account for around 1 in 5000 to 1 in 14 000 live births [3]. The etiology of atresia is an intrauterine vascular insult of the mesenteric vessels, resulting in multiple different types of atresia [4, 5]. There are primarily four types of atresia as described by Louw and later modified by Grosfeld [1]. Atresia classically involves complete obstruction of bowel, whether due to a luminal membrane, such as in Type 1, or a blind loop, such as in Types 2–4. Small bowel stenoses have also been described and referred to as a ‘Type 0’ atresia by Stollman *et al.* [6]. These types may be congenital or may be the result of a ruptured diaphragm of a type 1 atresia. The patient we presented above had a ‘Type 0’ atresia.

Classic atresias present with obstructive symptoms, such as oral intolerance, bilious emesis, abdominal distension, and sometimes delayed meconium passage. Early management principles for obstructive pathology in newborns include bowel decompression and fluid resuscitation, which were both initiated in our patient.

AXR is a useful diagnostic tool in early evaluation of intestinal atresia. Duodenal atresia, which has a different etiology than small bowel atresia, presents with the diagnostic "double bubble" on AXR. When the atresia is located more distally in the jejunum, the AXR will have more than two bubbles. The patient presented failed to have any of the classical findings of obstruction due to incomplete blockage of the intestine. The AXR shown in Fig. 2 clearly demonstrates a large amount of air passing through the intestine without an obvious area of obstruction.

Contrast studies are helpful and serve to rule out more urgent diagnoses such as malrotation with volvulus or Hirschsprung’s. UGI can diagnose patients with atresia if there is failure to pass contrast through to the colon, or the rectum if there is concern for colonic atresia. CE can help suggest an atresia if there is an underdeveloped or ‘small’ left colon; however, this is not always the case and can have another etiologies. The patient presented above had normal studies that showed contrast passing through without obstruction, and a normal caliber colon.

Surgical options for simple intestinal atresias include small bowel resection with primary end-to-end anastomosis. Proximal and distal bowel size discrepancies can be managed with various enteroplasties or end-to-oblique anastomosis with Cheatle technique [7, 8]. In conditions where there are perforation and frank contamination, the patient may require an enterostomy which would be reversed later.

Pre-operative and post-operative care should include nutritional support and continued bowel decompression. These can be achieved with early initiation of peripheral parenteral nutrition or TPN in infants unable to take oral feeds, and continuation of TPN post operatively while awaiting return of bowel function. Similarly, bowel decompression should be initiated early if there is concern for obstructive pathology and can be continued post operatively.

Surgical pathology interestingly revealed ileal stenosis with luminal narrowing. This would explain preoperative findings of evolving bowel gas distributions on imaging with intermittent abdominal distension and bowel dilation. The narrow opening intermittently allowed enough intraluminal air to pass through to create an evolving gas pattern that make the diagnosis difficult.

The prognosis for patients with atresias has improved significantly with the advent of TPN. Likewise operative mortality has declined significantly to as low as <1% [6, 9] and post-operative anastomotic leak rate between 5% and 8% [10]. The patient presented in this report recovered well despite the delay in diagnosis due to unusual clinical findings. This is a diagnosis that should be considered in patients who present with intermittent intestinal obstruction.
